# Supplementary material for: Predictive Values of Blood-Based RNA Signatures for the Gemcitabine Response in Advanced Pancreatic Cancer
Source: Cancers (Basel). 2020 Oct 30;12(11):3204. doi: 10.3390/cancers12113204 (PMC7692046; doi:10.3390/cancers12113204)
Supplement: Supplementary file 1 [file cancers-12-03204-s001.pdf]

# Supplementary Material: Predictive Values of Blood-Based RNA Signatures for the Gemcitabine Response in Advanced Pancreatic Cancer

**David Piquemal, Florian Noguier, Fabien Pierrat, Roman Bruno and Jerome Cros**

**Table S1.** Supplementary Materials and Methods.

| | Supplementary Methods |
|---|---|
| The assay was developed to meet to the MIQE guidelines and Clinical and Health Authorities' requirements. We established a complete analytical validation of the real-time PCR assay. This validation required a strict primer selection process and the following aspects and specifications have been addressed: | 1. Primers selection using Primer3Plus web tool (bioinformatics.nl/cgi-bin/primer3plus/primer3plus.cgi/).<br>2. In silico analysis of PCR primers:<br>A. First analysis of primers specificity using the UCSC Genome Bioinformatics Group of the University of California, Santa Cruz (In Silico PCR; http://genome.ucsc.edu/index.html). This study is completed using Primer-blast tool on The National Center for Biotechnology Information NCBI (ncbi.nlm.nih.gov);<br>B. Second analysis of primers specificity focused on interactions (hairpin, self-dimer or hetero-dimer) using the "OligoAnalyzer 3.1" web tool (http://eu.idtdna.com/analyzer/Applications/OligoAnalyzer/) to avoid important PCR equilibrium destabilizations;<br>C. Analysis of primers efficiency: The secondary structure of single stranded nucleic acids can be predicted using the Mfold software (http://mfold.rna.albany.edu/?q=mfold/download-mfold). By determining the secondary structure of single-stranded nucleic acids, it is possible to identify regions that are easily accessible by the primers to initiate DNA elongation; |
| Following this in silico study, we initiated in vitro analysis. | 1. In vitro analysis of PCR primers<br>A. First test of specificity via PCR product sizing, controlled using gel-based sizing by electrophoresis on agarose gel technique;<br>B. Second test of specificity via PCR product sequencing, controlled via the Sanger-based sequencing method;<br>C. Third test of specificity via negative controls, consisted in controlling possible cross reactivity of primers during the PCR process. For each gene, two negative controls have been used to highlight any cross reactivity of primers such as the formation of homo- or heterodimers, PCR contamination, genomic DNA contamination, etc:<br>A "NTC" control (no template control) which corresponds to a sample run in the exact same manner as the other Real-Time PCR reactions, but in which the cDNA matrix/template has been voluntary omitted. This control allows detecting any external contamination or other factors that could result in a non- specific increase in the fluorescence signal.<br>A "No RT" control (no reverse transcriptase control) which corresponds to a sample run in the exact same manner as the other Real-Time PCR reactions, but in which the cDNA matrix has been voluntary replaced by a RNA matrix.<br>D. Fourth test of specificity via control of amplicon Tm, consisted in controlling the melting temperature/dissociation temperature of the double-stranded DNA PCR product amplified. The Tm must be unique for each gene;<br>E. Test of efficiency: The efficiency of the RT-PCR amplification for each PCR reactions ranges between 88% and 103%, demonstrating that the PCR efficiencies are approved (http://miqe.gene-quantification.info). |
| Based on these controlled PCR parameters, we established analytical performance of the assay with definition of the limits of blank (LoB), the limits of detection (LoD) and the limits of quantification (LoQ), as well intra- and inter-patient variabilities. | |
| We concluded that given results obtained, both the repeatability and reproducibility of the Real-Time PCR assay have been confirmed. The analytical performance of the assay has thus been validated. | |
| Thus, we established a standard operating procedure (SOP) with 7 acceptance criteria, before the predictive value analysis of the signature: | 1. The LoB of NoRT controls must be validated;<br>2. The LoB of NTC controls must be validated;<br>3. All genes have to be measured (genes of interest *GOI* and housekeeping genes *HKG*);<br>4. Measured Cp value of each gene (*GOI* and *HKG*) is replicated 5 times and the measurement of a gene is accepted if at least 4 values are measured; |

| | |
|---|---|
| | 5. Repeatability of measures: Measured Cp value of each gene (*GOI* and *HKG*) is repeated 5 times and the measurement of a gene is accepted if at least 4 values are gathered to less than 0.2 Cp;<br>6. Limit of Quantification (LoQ): Measured Cp value of each gene (*GOI* and *HKG*) must be inferior to defined LoQ;<br>7. Temperature of melting (Tm): Measured Tm value of each gene (*GOI* and *HKG*) must be unique and closed to defined Tm values, with a maximal standard deviation of 0.5°C; |

**Table S2.** Overall survival clinical and biological data.

| **Baseline Characteristics** | **All Patients** | **GE − (OS)** | **GE + (OS)** | ***p*-value** |
|---|---|---|---|---|
| Number | 60 | 35 | 25 | |
| Gender (Female) | 24 (40%) | 11 (31%) | 12 (48%) | 1 |
| Age (years); median (range) | 62.7 (41.0–79.0) | 62 (41.0–78.0) | 65.0 (48.0–79.0) | 0.07 |
| Body mass index; mean (SD) | 24.2 (4.0) | 24.6 (3.6) | 23.7 (4.5) | 0.96 |
| Geographical region | | | | |
| France | 41 (68%) | 24 (68.5%) | 17 (68.0%) | |
| US | 12 (20%) | 7 (20%) | 5 (20%) | |
| Czech Rep | 4 (7%) | 3 (8.5%) | 1 (4%) | |
| Romania | 2 (3%) | 1 (3%) | 1 (4%) | |
| Poland | 1 (2%) | 0 (0%) | 1 (4%) | |
| CA 19–9 (U/mL); mean (SD) | 22369 (60803) | 32047 (74135) | 8821 (31221) | 0.153 |
| Albumin (g/L); mean (SD) | 35.7 (16.8) | 33 (8) | 38 (6) | 0.23 |
| QLQ-C30 Global; mean (SD) | 62.9 (18.6) | 67 (15) | 58 (14) | 0.16 |
| ECOG PS | | | | 0.04 |
| ECOG (0) | 18 (30%) | 9 (25%) | 9 (36%) | |
| ECOG (1) | 41 (68%) | 26 (75%) | 15 (60%) | |
| Monocyte count (per µL); median (range) | 0.55 (0.19–1.53) | 0.61 (0.2–1.53) | 0.53 (0.19–1.44) | 0.23 |
| Tumor localization‡ | | | | 0.4 |
| Head | 34 (57%) | 18 (51.4%) | 16 (64%) | |
| Body | 18 (30%) | 12 (34.2%) | 6 (24%) | |
| Tail | 17 (28%) | 14 (40%) | 3 (12%) | |
| Clinical stage | | | | 0.002 |
| Locally advanced | 14 (23%) | 4 (11%) | 10 (40%) | |
| Metastatic | 45 (75%) | 30 (86%) | 15 (60%) | |

Unless stated otherwise, data are the number of patients (%).The Fisher exact test or Chi-squared test was used for comparison of qualitative variables between OS + and OS − groups; analysis of variance was used for comparison of quantitative variables (Due to the low sample size we did not test for geographical region difference). ‡ Patients presenting tumors in more than one location are included in both categories. CA19–9; carbohydrate antigen 19–9. ECOG PS; Eastern Cooperative Oncology Group Performance Status. ECOG (0); ECOG score 0. ECOG (1); ECOG score 1. mITT; modified intent-to-treat population. All; All patients included. GE +; patient subgroup with a longer overall survival or progression free survival. GE −; patient subgroup with a shorter overall survival or progression free survival. QLQ-C30 Global; European Organization for Research and Treatment of Cancer (EORTC) quality of life questionnaire core 30 item global health status. SD; standard deviation. Note: The sum of the percentages might not be equal to 100%, and the sum of patients might not be equal to their total if the data were not available.

**Table S3.** Progression free survival clinical and biological data.

| **Baseline Characteristics** | **All Patients** | **GE − (PFS)** | **GE + (PFS)** | ***p*-value** |
|---|---|---|---|---|
| Number | 60 | 33 | 27 | |
| Gender (Female) | 24 (40%) | 11 (42%) | 14 (38%) | 0.23 |
| Age (years); median (range) | 62.7 (41.0–79.0) | 62.5 (43.0–79.0) | 62.9 (41.0–76.0) | 0.89 |
| Body mass index; mean (SD) | 24.2 (4.0) | 23.9 (3.5) | 24.6 (4.4) | 0.83 |
| Geographical region | | | | |
| France | 41 (68%) | 21 (64.5%) | 20 (72.5%) | |
| US | 12 (20%) | 9 (25%) | 3 (13.8%) | |
| Czech Rep | 4 (7%) | 2 (6.5%) | 2 (6.9%) | |
| Romania | 2 (3%) | 1 (4%) | 1 (3.4%) | |
| Poland | 1 (2%) | 0 (0%) | 1 (3.4%) | |
| CA 19-9 (U/mL); mean (SD) | 22369 (60803) | 12675 (36132) | 32732.4 (78589) | 0.51 |

| | | | | |
|---|---|---|---|---|
| Albumin (g/L); mean (SD) | 35.7 (16.8) | 35 (6) | 36 (9) | 0.16 |
| QLQ-C30 Global; mean (SD) | 62.9 (18.6) | 65.6 (15.7) | 60.2 (14) | 0.96 |
| ECOG PS | | | | 0.74 |
| ECOG (0) | 18 (30%) | 9 (25%) | 9 (34%) | |
| ECOG (1) | 41 (68%) | 24 (75%) | 17 (62%) | |
| Monocyte count (per μL); median (range) | 0.55 (0.19–1.53) | 0.6 (0.19–1.53) | 0.5 (0.2–1.44) | 0.06 |
| Tumor localization‡ | | | | 0.44 |
| Head | 34 (57%) | 18 (61%) | 16 (51.7%) | |
| Body | 22 (30%) | 14 (38.5%) | 8 (34.4%) | |
| Tail | 17 (28%) | 12 (35.5%) | 5 (20.6%) | |
| Clinical stage | | | | 0.06 |
| Locally advanced | 14 (23%) | 4 (16%) | 10 (31%) | |
| Metastatic | 45 (75%) | 28 (81%) | 17 (69%) | |

Unless stated otherwise, data are the number of patients (%).The Fisher exact test or Chi-squared test was used for comparison of qualitative variables between OS + and OS − groups; analysis of variance was used for comparison of quantitative variables (Due to the low sample size we did not test for geographical region difference). ‡ Patients presenting tumors in more than one location are included in both categories. CA19–9; carbohydrate antigen 19–9. ECOG PS; Eastern Cooperative Oncology Group Performance Status. ECOG (0); ECOG score 0. ECOG (1); ECOG score 1. mITT; modified intent-to-treat population. All; All patients included. GE +; patient subgroup with a longer overall survival or progression free survival. GE −; patient subgroup with a shorter overall survival or progression free survival. QLQ-C30 Global; European Organization for Research and Treatment of Cancer (EORTC) quality of life questionnaire core 30 item global health status. SD; standard deviation. Note: The sum of the percentages might not be equal to 100%, and the sum of patients might not be equal to their total if the data were not available.

**Publisher's Note:** MDPI stays neutral with regard to jurisdictional claims in published maps and institutional affiliations.

© 2020 by the authors. Licensee MDPI, Basel, Switzerland. This article is an open access article distributed under the terms and conditions of the Creative Commons Attribution (CC BY) license (http://creativecommons.org/licenses/by/4.0/).
